# The path to leadership: the career journey of academic health sciences library directors

**DOI:** 10.5195/jmla.2019.552

**Published:** 2019-01-01

**Authors:** Rick L. Fought, Mitsunori Misawa

**Affiliations:** Director, Health Sciences Library, University of Tennessee Health Science Center–Memphis, rfought1@uthsc.edu; Assistant Professor, Educational Psychology and Research–Adult Learning, University of Tennessee–Knoxville, mmisawa@utk.edu

## Abstract

**Objective:**

The authors examined the career journeys of academic health sciences library directors to better understand their leadership development and what led them to their leadership positions in libraries.

**Methods:**

A qualitative phenomenological approach was employed due to its focus on exploring and understanding the meaning that individuals ascribe to a particular phenomenon or experience. Eleven library directors from academic health sciences libraries at public universities with very high research activity agreed to participate in the study. The research question guiding this study was: What was the library directors’ career journey that led them into library leadership?

**Results:**

A major theme that emerged from the data was “Path to Leadership.” Although each participant’s journey was unique, common elements surfaced as they chronicled their careers that were informative as to how they understood their emergence and development as library leaders. The four categories defining this theme were breadth of experience, focused preparation, mentors, and recognition and development of leadership potential.

**Conclusions:**

Previous research suggests that leadership development and preparedness are important contributors to leadership effectiveness. It was encouraging to witness and understand the amount of preparation by participants to ready themselves for their roles as library directors. This study provides a comprehensive view of the path to library leadership that furthers understanding of the value of leadership development and preparedness and provides a model for aspiring library leaders.

## INTRODUCTION

Leadership development and preparedness are important contributors to leadership effectiveness [1]. Several studies have examined the leadership development of academic library directors and have evaluated how the library profession has promoted leadership development [2–8]. Most of this research has focused on mentoring, on-the-job training, library leadership training programs, and leadership development and preparedness in general. Lipscomb et al., for example, studied the National Library of Medicine (NLM)/Association of Academic Health Sciences Libraries (AAHSL) Leadership Fellows Program and found that study participants believed that the program helped individuals in their leadership development, contributed to the quality of leadership in the library profession, and improved succession planning and leadership development for AAHSL [5]. A few other studies focused on specific topics such as the necessity and value of a doctorate degree among small college library directors [9].

Each study has proved useful to understanding why leadership development and preparedness is important and what leadership skills a person should develop to prepare for a leadership position. Unfortunately, no library studies have provided a full picture that allows readers to understand how various elements of leadership development and preparedness interrelate and progress during one’s career journey. A cohesive picture of this career journey into library leadership would further understanding of the value of leadership development and preparedness and could serve as a model for those who are interested in moving into leadership positions at their libraries.

The authors specifically wanted to better understand the career journey of a library director at an academic health sciences library through examining the research participants’ experiences. We focused expressly on exploring the path and decisions that led them to leadership positions and how they prepared themselves to be effective leaders.

## METHODS

A qualitative phenomenological approach was employed in this study due to its emphasis on analyzing and understanding the personal experiences of academic health sciences library directors. The study concentrated on their perceptions and understanding of their leadership development and preparedness during their career journeys to becoming library directors. Qualitative phenomenological research was an appropriate design due to its focus on exploring and understanding the meaning that individuals ascribe to a particular phenomenon or experience [10]. Phenomenology attempts to get to the essence of a shared experience and works particularly well when studying phenomena that are difficult to quantify, such as leadership [11].

Study participants were selected using a purposeful sampling process, coupled with criterion-based sampling strategies. Purposeful sampling targets highly selective, information-rich cases or participants who are better able to explain and clarify the research questions [12]. For this study, the most appropriate participants were determined to be academic health sciences library directors. Criterion-based sampling strategies use predetermined criteria to eliminate participants who do not meet the criteria [12]. Participants met the following predetermined criterion: they were directors, or had equivalent titles, at public universities classified as research university/very high research activity (RU/VH) according to the US Carnegie Classification of Institutions of Higher Education. Health sciences libraries at these institutions are typically larger, have more employees, and potentially work with a broader and more complex range of issues than smaller institutions, which could yield a richer and more complete understanding of leadership at academic health sciences libraries. Additionally, the list of participants met the criterion of active AAHSL membership. An initial scan of universities meeting these criteria identified forty-eight institutions.

A purposeful sampling of fifteen academic health sciences library directors at these institutions were invited to participate in the study. These fifteen institutions were selected primarily due to their geographic locations to ensure all areas of the United States were included. Eleven participants accepted the invitation.

There is no exact determination of the number of participants that is adequate, as the intention of qualitative research is not to generalize the information but to explain participants’ specific personal experiences [10, 13]. However, Seidman recommends using two criteria to determine when a research study has a sufficient number participants [14]. The first criterion is sufficiency: are there enough participants in the study so that individuals outside the sample group can connect to the experience of those who are in it? There must to be a sufficient number of participants to reflect the wide range of individuals and contexts found in academic health sciences centers. The second criterion is saturation of information: do the researchers reach a point where they hear the same information from participants and no new information is being conveyed? At some point, a researcher no longer learns anything new, and at that point, any further data collection may be of limited value, although it is difficult to predetermine when this might occur [14]. Seidman, therefore, does not recommend a specific number of participants, as every researcher and every study is different, but does mention practical exigencies of time, money, and other resources as playing roles in determining the number of participants in a study [14]. We believe that both of Seidman’s criteria were met in regard to the number of participants in this study. Approval from the governing institutional review board was granted prior to contact with the participants in this study.

Data were collected primarily through phenomenological, semi-structured interviews. Two interviews were done with each participant, which allowed them time to “reconstruct and reflect upon their experience within the context of their lives” [14]. The interviews used primarily open-ended questions structured around the research question but allowed new ideas to be brought into the interviews, based on how participants responded. All interviews were conducted over the phone and were recorded to ensure accuracy and completeness. The interviews were later transcribed in preparation for coding.

Interview data were transcribed and analyzed using thematic analysis. Thematic analysis segregates data into groups with the use of codes or labels and searches for patterns that can be organized into broader categories. These categories are then constructed into themes that represent meaning from the data in response to the research questions [10, 15]. The order or combination of themes and categories for this study was dictated by the data. During analysis, certain ideas and perceptions stood out more than others, and their prominence was important to understanding the essence of this shared experience in leadership.

Qualitative research uses different standards of rigor than quantitative research to ensure trustworthiness. To a certain extent, the trustworthiness of a qualitative study depends on a thorough and rigorous research design [10]. This study also used member checking, peer debriefing, and triangulation to ensure its trustworthiness. In member checking, the researcher gives participants the opportunity to explain any of their interview answers and comment on whether their interviews accurately and fully reflect their experience of the phenomenon under study [13]. Peer debriefing is an external review of the research process done for the researcher by a peer who provides an independent viewpoint [13]. Finally, triangulation refers to the use of multiple data collection methods and/or multiple data sources to substantiate the research findings [10, 15].

Subjectivity or bias is unavoidable when conducting research. It is incumbent on researchers to acknowledge this and to attempt to identify their subjectivities, assumptions, and stereotypes throughout the course of their research [15]. Although acknowledgment of this subjectivity or bias does not eliminate it, it allows researchers to manage and minimize the impact on the research process and achieve a better understanding of themselves [15]. In this study, one researcher is an experienced librarian in a leadership position at an academic health sciences library and the other is a professor of education with no prior work in libraries. Both are very interested in the study of leadership effectiveness in higher education.

## RESULTS

After analyzing the data gathered from this research question, we determined that one major theme emerged with four categories defining the theme. The theme was labeled “Path to Leadership,” and its four categories were breadth of experience, focused preparation, mentors, and recognition and development of leadership potential. Pseudonyms are used to protect the identities of the research participants.

### Breadth of experience

Several participants communicated during their interviews how the position of library director required a broad perspective and an understanding of the big picture of how academic health sciences libraries work and how they fit in at their institutions—now and in the future. They believed that this was critical for a director to be effective and do their job well. As they spoke about their individual paths to their positions of leadership, the participants’ variation in education and work experiences was remarkable, including one participant with a master’s in business administration and another with a law degree. For instance, Patricia worked in a variety of positions before becoming a director, each of which provided her with valuable experience that prepared her well for her future role:

I have worked across like all areas of the library. I was a cataloger for a year. I worked in technical services for a year. I was an assistant director. I was head of a small library, small two-, three-person library. I dealt in such a wide range of health sciences from medical education to medical research to healthcare policy. I really think that breadth of exposure in those three very different institutions I worked at gave me a broad perspective, an ability to detect, not quite the right word, but to suss [out] what’s really critical, what’s really good practice, and what’s just local custom. And I felt really prepared when I came to [my current position] for the challenge that I was given here.

Another important aspect of this category pertained to opportunities for growth and experience given to participants at various stages of their careers that allowed them to develop their leadership skills and learn important lessons regarding how academic health sciences libraries work. For example, Terri spoke about opportunities she was given that she thought were instrumental in her personal leadership development:

I was given an opportunity to organize a conference for the whole campus. It was going to be a technology expo. I was given stretch assignments and conference event planning exercises. There was another opportunity [at my library] where I had to decommission all photocopiers and move us to a printing card system. I also had to move us from a home-grown system to a commercial-based library integrated system. We went from a home-grown system to Innovative Interfaces, Inc. So, I guess, I was given a lot of implementation assignments. I had to do the research or was part of the team that did the research. But I just remember those being really risky, fun, rewarding opportunities that really challenged me and kept me motivated, and I appreciated the fact that those directors really were willing to take a risk on me.

It was during this time of growth that participants said they started thinking that they could be directors at some point in their future and initiated a more deliberate plan of preparation to help them achieve that goal.

### Focused preparation

Once they set the goal of becoming an academic health sciences director, many participants took a more focused and planned approach to preparing themselves for that position. They had been exposed to enough leadership opportunities that they better understood where they needed to develop their skills and gain more experience. Many participants took advantage of opportunities that their universities and/or professional organizations provided to develop their leadership abilities. Olivia talked about specific leadership skills that she sought to develop to provide herself with a solid foundation before becoming director. Specifically, she mentioned leadership classes on hiring employees, budgeting, and interpersonal relationships.

Several specific leadership programs were mentioned by multiple participants as being particularly helpful in their development as leaders. Each participant took something different from the programs, but all of them found the experience valuable and an important step in their paths toward becoming a library director. For some, the experience served, among other things, as a confidence booster. One research participant, Karen, had this to say:

One thing that helped me gain more confidence was getting the opportunity to attend the Bryn Mawr Summer Institute for Women in Higher Education Administration. Those kinds of opportunities I thought were really helpful not only in building skills and perspective, but also providing a little bit of a jolt or booster. Morale booster as well as a knowledge booster. I had also gone to the Harvard/[Association of College and Research Libraries] ACRL Leadership Institute for Academic Librarians. While Bryn Mawr was three weeks and a half and residential *[chuckles]*, ACRL was one week and residential. But what was packed into that week was just incredible. I think the thing that I really liked the best about it was learning about the four frames because it helped me get even a quicker way of understanding what’s going on around me and how to interpret things. I found that very valuable.

The main program mentioned by participants regarding their leadership development was the NLM/AAHSL Leadership Fellows Program, a yearlong intensive leadership program designed to prepare emerging leaders for the position of library director in academic health sciences libraries [5]. Several research participants had been part of this program and spoke well of its contribution to their development as leaders in academic health sciences libraries. Debra said:

So basically, doing the AAHSL Leadership Fellow Program, the one-year leadership institute, was really good for me. It helped reframe many things I had been doing and gave them a name and then I understood what it was I had been doing. And going forward I felt more empowered with that knowledge, knowing what I needed to do. And I do a lot of leadership [continuing education courses] CEs, I read a lot, I have a vast collection of supervisory manuals. I also go to the CEs here at [my university] where they offer a 6-month leadership training class, supervisory training class. [My alma mater] had a supervisory training class that lasted 6 weeks. So, I’ve been through all the training courses at [my alma mater] and [my university], learning as much as I possibly could and applying as much as I possibly can in the role I currently have.

The research participants dedicated countless hours toward the singular task of developing their leadership abilities to add to their already solid foundations of knowledge and skills regarding libraries in general. Once they decided to take that next step in their careers, they understood the need for additional knowledge and experience to be successful and then selectively sought out that knowledge and experience.

### Mentors

Another important part of leadership development mentioned by several participants was mentors who helped them at various stages of their careers. Mentor relationships were described by many as being valuable in advancing the participants’ careers. Mentors not only educated participants about the demands of leadership at academic health sciences libraries, but also served as sounding boards for ideas and advice once the participants began moving into leadership positions. When asked about what made the biggest difference in her development as a leader, Mary Lou replied:

I think having a boss who acted as a mentor and I was very, very fortunate to have had an excellent boss who was also a very good mentor. She would challenge me to think differently about how I might approach a problem, and she would let me sit in with her on certain leadership opportunities. I think having a boss and a mentor who sees opportunities and then can match those opportunities to who you are as person, are very important. And she had a gift to be able to do that. She has not only done that with me, she’s done it with several others. And so that really helped to form my experiences as a library director.

Jill and Terri mentioned seeking out mentors who modeled the behaviors that they thought were effective and were on the career paths they hoped to follow. These mentors were considered to have the types of knowledge, skills, and experiences that they were seeking. In turn, these mentors went beyond imparting advice and wisdom and directly helped the participants advance their careers. Terri mentioned, “I had great mentors along the way and some of my mentors were always watching out for me for [the] next career opportunities.”

Almost every research participant mentioned a mentoring relationship that they had experienced somewhere during their paths to becoming a library director. In each case, they spoke about this mentor relationship in reverent terms and expressed a desire to mentor librarians themselves. They deeply appreciated the mentoring that they received and wanted to give that same experience back to the next generation of library leaders.

### Recognition and development of leadership potential

The last category for this theme provided a deeper look at how participants recognized and developed their own leadership potential as well as how they recognized emerging leaders in the library profession and what they did to assist in developing that leadership potential. Richard said that what he looked for in potential library leaders was:

I know the big one I looked for, and that was people that could see a big picture. People that didn’t focus so narrowly on their own set of duties that they couldn’t grasp the way the whole system worked. They had to know their role in our library system in the library here but boy, if you can’t see how that fits into a larger picture, you’re sort of doomed. And I have seen people tripped up by that, who just won’t pull back and see the bigger picture. Now, how I know if they’re seeing the bigger picture or not, I don’t know. I guess what the things they’re interested in, what kind of projects they take on, even the way they develop relationships with other faculty within the library and within our constituent groups, I guess. So, I guess that’s what I’m looking for, the way they build relationships and the way they sort of build their worldview.

Several participants mentioned being enthusiastic, having a good attitude, and being able to move things forward as good signs of leadership potential. Lily described people with leadership potential as overachievers who always tried to exceed expectations. Dean made similar comments regarding initiative and high expectations. It was clear from the interviews that many directors wanted people who had the stamina, energy, and passion to perform at what experience had told them was a highly demanding position that has a tremendous impact on its institution.

In terms of how these directors helped someone they thought showed leadership potential, many participants would give them projects as opportunities to develop their leadership potential. Hannah spoke of giving her staff increasingly large projects as they proved themselves capable of handling the work and responsibility. She described her process like this:

I give people smaller projects and see how they do, and then give them bigger projects and watch them succeed. And I keep an eye on them and not just throw them in the deep end, but try to coach them in how to succeed, I think. People that can listen, benefit from the coaching. Some people, you can tell them, but they just don’t get it. So, people who have good judgment and a good attitude, you can teach them what they need to know in terms of skills.

Each participant seemed especially excited to talk about their efforts to help, encourage, and develop new leaders in academic health sciences libraries. In discussions with participants about their experiences along the career path that led them to becoming library directors, it was clear that they did not do it alone. They benefited greatly from mentors, leadership development programs offered by library professional organizations, and many opportunities given them by supervisors over the course of their careers. In this spirit, they were eager to do the same for others and seemed to enjoy passing on the lessons that they learned along their journeys, perhaps more than any other part of their experience as library directors.

## DISCUSSION

We sought to better understand the career journeys of library directors at academic health sciences libraries through examining research participants’ experiences. Specifically, we were interested in the preparation and path that led participants to the leadership positions they currently held at their libraries. Our findings were consistent with most other research on leadership development in academic libraries. However, whereas previous studies concentrated mostly on a particular aspect of leadership development, such as the most desirable traits for library directors or mentorship, we examined the larger process of preparing to become a library director [16, 17]. For example, all directors in the study mentioned their prior library experience, specific leadership training programs, and/or mentor relationships as being critical to their preparedness as library directors. The participants also mentioned being active in several professional library organizations. This supported the findings of O’Keeffe et al.’s survey showing that directors prepared themselves for the position through education attainment, prior experience, and professional activities [18]. O’Keeffe et al., however, did not address mentors or how these directors recognized and developed their leadership potential.

Most research participants spoke directly about mentoring relationships they had throughout their careers that were beneficial to them. Bonnette and Kirkland studied the benefits of mentors to minorities and women, respectively, in attaining leadership positions in academic libraries [2, 19], while Mavrinac made a case for peer mentoring to foster leadership development [16]. Together, the present and previous studies suggest that mentoring is valuable to leadership development and produces positive outcomes. Several of our research participants expressed a desire to serve as mentors for potential new library leaders, illustrating the value of these relationships.

Many of our research participants were involved with the NLM/AAHSL Leadership Fellows Program. As previously mentioned, this one-year program is designed to prepare emerging leaders for the position of library director in academic health sciences libraries [5]. Debra mentioned her experience with the program as being one of the most useful activities for preparing herself to become a library director. Terri and Lily both discussed how important going through the program was for their careers and that it was a capstone experience that brought many academic health sciences library leadership concepts together for them. When Lipscomb et al. reviewed the program by interviewing past fellow graduates, they found that the program enhanced leadership skills, provided fellows a network of peers, and gave them credibility as candidates for library director positions [5]. Discussions with research participants who took part in the program confirmed these findings.

The implications of this study are significant for librarians who are considering a move into leadership positions. While every research participant’s journey to leadership was unique, there were common themes that emerged that can serve as a model or guide for someone who is thinking about becoming a library director, particularly in an academic health sciences library. For librarians who are already in leadership positions, this study could better enable them to identify those people with leadership potential and guide them as to how to develop that potential. As libraries currently face challenging times, developing effective leadership is important to our future success [20].

There are notable limitations of this study that bear mentioning. Collecting data, including the two phenomenologically based semi-structured interviews, and completing the transcription and analysis of the data were completed in approximately three months. The second interview with each participant was conducted approximately one week after the first interview. Although this three-month period for data collection and analysis was adequate, we would have benefited from more time for analysis. It would have been better to have scheduled visits to all of the participants’ libraries. Also, the interviews typically lasted between thirty and sixty minutes, and more depth and detail could have been gained by extending the interviews to sixty and ninety minutes, if the participants’ schedules allowed.

Future research in this area could expand to investigating new library directors to better understand the challenges that they face and how well they are meeting those challenges. As important as it is to support and develop librarians who are aspiring to become leaders, it is equally important to continue encouraging and developing their leadership potential after they become library directors. Also, there continues to be a need to sufficiently measure library directors’ effectiveness [21]. Finally, it would be interesting to explore whether or how the experiences of academic health sciences library directors might differ from traditional academic or hospital library directors.

We sought to better understand the career journeys of library directors at academic health sciences libraries and how they prepared themselves to be effective leaders. We identified a theme from the data, “Path to Leadership,” with four categories that distilled the essence of the participants’ experiences as they developed and prepared themselves for effective leadership. These four categories—breadth of experience, focused preparation, mentors, and recognition and development of leadership potential—can guide librarians who are considering leadership positions in their careers and guide current library directors who are interested in identifying and developing librarians with leadership potential. The study also provides a comprehensive view of the path to library leadership that furthers understanding of the value of leadership development and preparedness.
